# Supplementary material for: Effect of Oats and Wheat Genotype on In Vitro Gas Production Kinetics of Straw
Source: Animals (Basel). 2021 May 26;11(6):1552. doi: 10.3390/ani11061552 (PMC8228468; doi:10.3390/ani11061552)
Supplement: Supplementary file 1 [file animals-11-01552-s001.zip › animals-1163521-supplementary.pdf]

animals MDPI

Article

Supplementary Materials: Effect of Oats and Wheat Genotype on In Vitro Gas Production Kinetics of Straw

Karen A. Peñailillo, Maria Fernanda Aedo, Maria Carolina Scorcione, Mónica L. Mathias, Claudio Jobet, Manuel Vial, Iris A. Lobos, Rodolfo C. Saldaña, Paul Escobar-Bahamondes, Paulina Etcheverría and Emilio M. Ungerfeld

Table S1. Effect of oats and wheat genotypes from three breeding trials (Oats 1, advanced breeding lines and commercial varieties; Oats 2, commercial and historical varieties; Wheat, advanced breeding lines and commercial varieties) on straw morphology.

Breeding trial	Response	Overall mean	SEM [1]	Min	Max	Genotype p =
Oats 1	$av\Phi$ [2] (mm)	5.63	0.120	4.93	6.47	0.002
	$R\Phi$ (mm/mm)	0.910	0.0118	0.891	0.942	0.049
	%Leaves (g/100 g)	26.1	0.931	20.6	29.7	0.003
	δ (g/cm^3)	0.125	0.00387	0.108	0.145	0.004
Oats 2	$av\Phi$ (mm)	5.10	0.103	3.94	6.31	0.001
	RΦ (mm/mm)	0.904	0.0110	0.885	0.925	0.060
	%Leaves (g/100 g)	24.4	0.948	18.1	27.3	0.004
	δ (g/cm^3)	0.122	0.00414	0.0947	0.177	0.001
Wheat	$av\Phi$ (mm)	4.44	0.0559	4.13	4.74	0.002
	$R\Phi$ (mm/mm)	0.962	0.00813	0.920	0.985	0.008
	%Leaves (g/100 g)	31.7	0.964	28.3	37.6	0.003
	δ (g/cm^3)	0.128	0.00446	0.0919	0.148	0.002

[1] SEM: standard error of the mean, Min: minimum, Max: maximum; [2] $av\Phi$ = Average diameter of the first and second internodes; $R\Phi$ = Diameter of the first internode / diameter of the second internode; *%Leaves* = percentage of leaves by mass; δ = apparent density.

Table S2. Prediction of in vitro dry matter digestibility form gas production at 8, 24 and 40 h of incubation and from the theoretical maximum and the fractional rate of gas production.

Predictor	Regression equation
P_8 (mmol/g DM incubated) [1]	$IVDMD = -18.4(\pm 9.57; p = 0.059) + 36.7(\pm 5.29; p < 0.001)x; R2 = 0.40(p < 0.001)$
P_{24} (mmol/g DM incubated)	$IVDMD = -35.7(\pm 10.3; p < 0.001) + 26.7(\pm 3.29; p < 0.001)x; R2 = 0.48(p < 0.001)$
P_{40} (mmol/g DM incubated)	$IVDMD = -23.7(\pm 9.34; p = 0.013) + 19.2(\pm 2.50; p < 0.001)x; R2 = 0.45(p < 0.001)$
P_{max} (mmol/g DM incubated)	$IVDMD = -3.17(\pm 7.67; p = 0.68) + 12.0(\pm 1.79; p < 0.001)x; R2 = 0.39(p < 0.001)$
c (h^{-1})	$IVDMD = 62.5(\pm 5.15; p < 0.001) - 285(\pm 101; p = 0.006); R2 = 0.10(p = 0.006)$

[1] P_8: gas production at 8 h of incubation, P_{24}: gas production at 24 h of incubation, P_{max}: theoretically maximal gas production, c: fractional rate of gas production, IVDMD: in vitro apparent digestibility of dry matter, expressed as a percentage of dry matter incubated.

Table S3. Coefficients of determination between agronomic traits and gas production kinetic parameters of straw from three genetic improvement trials (Oats 1, advanced breeding lines and commercial varieties; Oats 2, commercial and historical varieties; Wheat, advanced breeding lines and commercial varieties) incubated in rumen in vitro cultures.

Breeding trial	Gas production kinetics parameter	Grain yield (ton/ha)	Lodging incidence (%area)	Lodging severity (1–5)	Halo blight (%)	Barley yellow dwarf virus (N)	Crown rust (%)
Oats 1	P_8 (mmol/g DM incubated) [1]	0.010 [2] (p = 0.64)	0.21 (p = 0.024)	0.23 (p = 0.019)	0.0020 (p = 0.84)	0.025 (p = 0.46)	0.072 (p = 0.20)
	P_{24} (mmol/g DM incubated)	0.077 (p = 0.19)	0.097 (p = 0.14)	0.080 (p = 0.18)	0.015 (p = 0.57)	0.037 (p = 0.37)	0.044 (p = 0.33)
	P_{40} (mmol/g DM incubated)	0.085 (p = 0.17)	0.062 (p = 0.24)	0.038 (p = 0.36)	0.017 (p = 0.54)	0.044 (p = 0.33)	0.044 (p = 0.33)
	P_{max} (mmol/g DM incubated)	0.057 (p = 0.26)	0.030 (p = 0.42)	0.0078 (p = 0.68)	0.010 (p = 0.64)	0.051 (p = 0.29)	0.0078 (p = 0.68)
	c (h^{-1})	0.011 (p = 0.62)	0.0018 (p = 0.84)	0.031 (p = 0.41)	0.019 (p = 0.52)	0.024 (p = 0.47)	0.022 (p = 0.49)
Oats 2	P_8 (mmol/g DM incubated)	0.085 (p = 0.16)	0.10 (p = 0.12)	0.061 (p = 0.23)	0.00084 (p = 0.89)	0.000043 (p = 0.98)	- [3]
	P_{24} (mmol/g DM incubated)	0.10 (p = 0.12)	0.15 (p = 0.053)	0.069 (p = 0.21)	0.0021 (p = 0.83)	0.000022 (p = 0.98)	-
	P_{40} (mmol/g DM incubated)	0.085 (p = 0.16)	0.14 (p = 0.070)	0.045 (p = 0.31)	0.0059 (p = 0.71)	0.00098 (p = 0.88)	-
	P_{max} (mmol/g DM incubated)	0.034 (p = 0.38)	0.071 (p = 0.20)	0.0075 (p = 0.68)	0.018 (p = 0.52)	0.0064 (p = 0.70)	-
	c (h^{-1})	0.044 (p = 0.31)	0.050 (p = 0.28)	0.12 (p = 0.096)	0.038 (p = 0.35)	0.045 (p = 0.31)	-
Wheat	P_8 (mmol/g DM incubated)	0.14 (p = 0.076)	-	-	-	-	-
	P_{24} (mmol/g DM incubated)	0.11 (p = 0.12)	-	-	-	-	-
	P_{40} (mmol/g DM incubated)	0.063 (p = 0.24)	-	-	-	-	-
	P_{max} (mmol/g DM incubated)	< 0.010 (p = 0.90)	-	-	-	-	-
	c (h^{-1})	0.0084 (p = 0.67)	-	-	-	-	-

[1] P_8: gas production at 8 h of incubation, P_{24}: gas production at 24 h of incubation, P_{40}: gas production at 40 h of incubation, P_{max}: theoretically maximal gas production, c: gas production rate; [2] R^2 value.; [3] -: not determined.
